# The impact of COVID-19 on the management and outcomes of patients with proximal femoral fractures: a multi-centre study of 580 patients

**DOI:** 10.1186/s13018-021-02301-z

**Published:** 2021-02-24

**Authors:** Alice Wignall, Vasileios Giannoudis, Chiranjit De, Andrea Jimenez, Simon Sturdee, Sohail Nisar, Hemant Pandit, Aashish Gulati, Jeya Palan

**Affiliations:** 1 Leeds Teaching Hospitals NHS Trust, Leeds, UK; 2 Huddersfield Royal Infirmary, Huddersfield, UK; 3 Sandwell & West Birmingham Hospitals NHS Trust, Lyndon, UK

**Keywords:** Proximal femoral (hip) fracture, COVID-19, Intracapsular hip fracture, Extracapsular hip fracture, 30-day mortality, Time to theatre, Length of stay

## Abstract

**Background:**

On the 11th March 2020, the World Health Organization declared the COVID-19 outbreak a pandemic. Multiple new guidelines were proposed and existing models of social, domestic and hospital care altered. Most healthcare systems were largely unprepared for this, and the pandemic has tested their adaptability. This study aimed to assess the impact of COVID-19 on the demographics, presentation, clinical management and outcomes of patients with proximal femoral (hip) fractures comparing them to a similar cohort of patients admitted a year earlier.

**Methods:**

This retrospective multi-centre cohort study compared all patients admitted with hip fractures between 1st March and 30th May 2019 (group PC: pre-COVID-19) with hip fracture patients admitted over the same time period during the pandemic in 2020 (group C: COVID-19). The data was obtained from the hospitals’ local and National Hip Fracture Databases. Mortality data was checked with the Office for National Statistics (ONS). Primary outcomes were time to theatre, in-patient length of stay and 30-day mortality.

**Results:**

A total of 580 patients were included (304 group PC, 276 group C). Patient demographics including Charlson Comorbidity Index and Nottingham Hip Fracture Scores were broadly similar across the two cohorts. There was a significant reduction in the percentage of total hip replacements (11 to 5%, *p* = 0.006) in group C. There was an increase in conservative management (1 to 5%, *p* = 0.002) in group C. Time to theatre was significantly delayed in group C (43.7 h) vs group PC (34.6 h) (*p* ≤ 0.001). The overall length of hospital stay was significantly longer in group PC (16.6 days) vs group C (15 days) (*p* = 0.025). The 30-day mortality rate in group C was 9.8% compared to 8.2% in group PC (*p* = 0.746), but for COVID-19 (+) patients, it was significantly higher at 38.2% vs 5.8% in COVID-19 (−) patients (*p* < 0.001).

**Conclusion:**

This is one of the largest multi-centre comparative cohort study in the literature to date examining the impact of the COVID-19 pandemic on the management of hip fracture patients. Whilst mortality rates were similar in both groups, COVID-19-positive patients were almost seven times more likely to die, reflecting the seriousness of the COVID-19 infection and its sequelae in such elderly, vulnerable patients.

## Background

Severe acute respiratory syndrome coronavirus 2 (SARS-CoV-2) has spread to most countries in the world, with the World Health Organization (WHO) declaring a COVID-19 pandemic on March 11, 2020 [1]. This has placed enormous strains on National Health Systems (NHS), leading to significant changes in the way many conditions are managed [2–7]. In the United Kingdom (UK), this has led to unprecedented pressures on the NHS [8]. Even during this pandemic, patients with hip fractures remain one of the commonest reasons for admissions [9–11]. Over 60,000 hip fractures occur annually and represent a significant proportion of the trauma workload for most hospitals [12]. Patients with hip fractures are usually frail and elderly with multiple pre-existing co-morbidities [13, 14]. Expedited surgery, usually in the form of fixation, a hemiarthroplasty or total hip replacement, is usually indicated for most patients, as this is associated with better outcomes for patients [15, 16]. Although almost all planned surgery has been cancelled in the UK during this pandemic [17], the incidence of hip fractures requiring emergency surgical intervention during this period has remained constant [9–11]. The British Orthopaedic Association (BOA) issued guidance on how to manage trauma patients in the COVID-19 situation. They stated that “surgeons will need to consider alternative ways to manage many aspects of urgent orthopaedic conditions and trauma. Changes to standard management plans may be required to minimise patient exposure to disease and overall impact on resources” [18].

Our understanding of the impact of COVID-19 on this population of patients remains limited with a paucity of clinical data on this subject. An international collaborative study looked at the mortality rates of patients that were COIVD-19-positive (7 days prior or 30 days after elective or emergency surgery) and found that the overall 30-day mortality rate was 23.8%. Breaking this down, mortality rates for emergency surgery in these patients were statistically significantly higher at 25.6% (214/835) vs elective surgery at 18.9% (53/280). When defining the surgery by specialty (combining elective and emergency cases), the COVID-19-positive patients undergoing orthopaedic surgery had the 5th highest mortality rate out of 15 specialties at 28.8% (86/299) [19]. Another recently published multi-centre retrospective cohort study from London comparing COVID-19 positive and negative hip fracture patients found an increased 30-day mortality rate of 30.5% (25/82) vs 10.3% (35/340), respectively (*p* < 0.001) [20].

In response to the COVID-19 pandemic, many hospitals have had to reduce theatre capacity significantly and to run so-called ‘hot’ and ‘cold’ theatres to accommodate patients who were COVID-19 positive and negative, respectively. Dedicated trauma lists have stopped due to redeployment of anaesthetic and theatre staff. Anecdotally, the time taken to undertake surgery has increased due to logistical challenges and the need to minimise the risk of spreading the virus in a high-risk environment where aerosolised particles are generated [3, 21–26]. These changes during unprecedented times are likely to have a significant impact on the ability to deliver optimal hip fracture care which can impact patient outcomes.

The aim of this multi-centre study was to assess the impact of COVID-19 on the demographics, presentation, clinical management and outcomes of patients with proximal femoral (hip) fractures comparing them to a similar cohort of patients admitted a year earlier. This, to our knowledge, is one of the largest studies of its kind to assess the impact of the COVID-19 pandemic on hip fracture trauma services in England and provides vital information for the continued management of these patients in the current situation and to help prepare for future pandemics.

## Methods

A multi-centre retrospective observational study was undertaken in three hospitals in England, which included two large district general hospitals (DGH) and one major trauma centre (MTC). One of the DGH (hospital A) was based in the West Midlands, with the other DGH (hospital B) and the MTC (hospital C) based in Yorkshire. In total, the three hospitals serve a population of over 1.8 million people.

This study was registered with each of the hospitals as a service evaluation study and compared all patients admitted with proximal femoral fractures between 1st March and 30th May in 2019 (group PC: pre-COVID-19) to patients of the same diagnosis admitted over the same time period during the pandemic in 2020 (group C: COVID-19). The data was obtained from the hospitals’ local and National Hip Fracture Databases (NHFD). Mortality data was checked with the Office for National Statistics (ONS). The primary outcomes were 30-day mortality, time to theatre and in-patient length of stay.

Patients were tested for COVID-19 with throat and nose swab assays using reverse transcriptase polymerase chain reaction (RT-PCR) for SARS-CoV-2. In hospital B, all patients were tested. Initially, patients admitted to hospitals A and C were only tested for COVID-19 if there were indications to do so according to local hospital guidelines: presence of new cough, fever (> 37.8°), acute respiratory infection, acute worsening of underlying respiratory illness and COVID-19 radiological changes (bilateral CXR infiltrates). However, by the end of April, both of these hospitals were testing all patients on admission for COVID-19 as per hospital protocols. Overall, 58% (46/79) and 83% (111/134) of patients in hospital A and C, respectively, were tested for COVID-19. Patients were therefore classified as COVID-19 negative if they were asymptomatic or had a negative throat and nose swab. Patients were classified as COVID-19 positive in the presence of clinical symptoms and/or a positive throat and nose swab at any time point during their in-hospital stay. Repeat swab samples were performed in patients with negative swab assay results with persistent or new suspicious clinical features. The minimum interval between swab samples was 24 h, and all swab samples were obtained by local nursing staff.

The following data were recorded for all patients: patient demographics and baseline characteristics (age, gender, laterality of surgery, American Society of Anesthesiologists (ASA) grade), preoperative factors (Charlson Comorbidities Index (CCI), Nottingham Hip Fracture Score (NHFS), mobility status, social situation, comorbidities), operative factors (fracture type, operative intervention performed) and patient outcomes (time to theatre, mortality at 30 days, length of hospital stay). Additionally, for patients in group C, the presence of COVID-19 symptoms and RT-PCR SARS-CoV-2 swab results (if taken) were noted.

General descriptive analysis was performed for both time periods in groups PC and C. Continuous variables were reported using means and standard deviations (SD) if they followed a normal distribution; otherwise, the median and range were used. Absolute frequencies and proportions (as a percentage) were used to summarise categorical variables. To compare the two groups, where the data was parametric, the Students *t* test and Pearson chi-squared analysis were employed. Where the data was nonparametric, the Mann-Whitney *U* test was applied. For all analyses, the statistical package STATA version 13.1 (StataCorp. 2013. Stata Statistical Software: Release 13. College Station, TX: StataCorp LP) was used. *p* value of 0.05 assumed to be significant.

## Results

There were 580 patients included in this study, which comprised 304 patients in group PC and 276 patients in group C. There was an average of 3 patients admitted per day in both group PC (range 1–14 patients per day) and group C (range 1–10 patients per day). Patient demographics including gender, CCI, NHFS and fracture type were similar across the two cohorts. There were less ASA 3 but more ASA 4 patients in group C compared to group PC. (see Table 1).

**Table 1:** Patient demographics in the pre-COVID-19 (PC) and COVID-19 (C) groups

Variable	PC (*n* = 304)	C (*n* = 276)	*p* value
Age, years, mean (SD)	82.4 (10.5)	81.3 (11.2)	0.243
Gender %, M:F	48.7:54.3	51.3:45.7	0.201
ASA, *n* (%)			
1	6 (2.0)	4 (1.5)	0.628
2	71 (23.4)	74 (26.8)	0.337
3	189 (62.2)	140 (50.7)	0.005
4	37 (12.2)	57 (20.7)	0.006
5	1 (0.3)	1 (0.4)	0.945
CCI, mean (SD)	5.5 (2.3)	5.2 (2.1)	0.332
NHFS, mean (SD)	4.7 (1.5)	4.9 (1.7)	0.048
Fracture diagnosis, *n* (%)			
Extracapsular	136 (44.7)	110 (39.9)	0.235
Intracapsular	149 (49.0)	148 (53.6)	0.267
Subtrochanteric	17 (5.6)	6 (2.2)	< 0.001
Periprosthetic	2 (0.66)	8 (2.9)	< 0.001
Others	0	4 (1.5)	< 0.001

*SD* standard deviation, *ASA* American Society for Anaesthesiology, *M* male, *F* female, *CCI* Charlson Comorbidity Index, *NHFS* Nottingham Hip Fracture Score

Table 2 compares the operations and outcomes of the two groups. There were no significant differences in the numbers of dynamic hip screw (DHS) (*p* = 0.770) and hemiarthroplasties (*p* = 0.363) performed. In group C, there was a significant reduction in the percentage of total hip replacements (THR) (11 to 5%, *p* = 0.006) and an increase in patients managed conservatively (1 to 5%, *p* = 0.002). The time to theatre (in hours) was significantly longer in group C (43.7) vs group PC (34.6) (*p* < 0.001). Length of hospital stay was significantly longer in group PC (16.6 days) vs group C (15 days) (*p* = 0.025). The 30-day mortality rate was 9.8% in group C vs 8.2% in group PC (*p* = 0.746).

**Table 2:** Procedures and outcomes in the pre-COVID-19 (PC) and COVID-19 (C) groups

Variable	PC (*n* = 304)	C (*n* = 276)	*p* value
Operation procedure, *n* (%)			
DHS	75 (24.7)	71 (25.7)	0.770
Hemiarthroplasty	110 (36.2)	110 (39.9)	0.363
THR	33 (10.9)	13 (4.7)	0.006
IM nail	79 (26.0)	55 (19.9)	0.084
ORIF	0	4 (1.5)	0.035
CHS	4 (1.3)	8 (2.9)	0.181
Conservative	3 (1.0)	15 (5.4)	0.002
Time to surgery, h, mean (SD)	34.6 (29.6)	43.7 (34.6)	< 0.001
Length of stay, days, mean (SD)	16.6 (10.6)	15.0 (10.6)	0.025
Mortality, *n* (%)	25 (8.2)	27 (9.8)	0.7456

*DHS* dynamic hip screw, *THR* total hip replacement, *IM* intramedullary, *ORIF* open reduction internal fixation, *CHS* cannulated hip screws, *SD* standard deviation

Table 3 shows the patient demographics and outcomes between COVID-19-positive and COVID-19-negative patients in group C. The incidence of COVID-19 was 12.3% (34/276). Within this group, 35% (12/34) were positive for COVID-19 on admission, 27% (9/34) acquired COVID-19 whilst an inpatient (either the first swab was negative or they were positive > 14 days post-admission) and 9% (3/34) tested positive post-admission. The remaining 29% (10/34) had positive swabs within 14 days of admission and no admission swab; therefore, it is unknown as to whether these patients were positive on admission or acquired COVID-19 whilst an inpatient. For COVID-19-positive patients, the 30-day mortality rate was significantly higher at 38.2% vs 5.8% in COVID-19-negative patients (*p* < 0.001). The COVID-19-positive patients were older and had a higher NHFS. The time taken to have surgery was also delayed further in the COVID-19-positive patients at a mean of almost 55 h, and the mean length of stay was also significantly longer (17.6 days).

**Table 3:** Comparing COVID-19-positive vs COVID-19-negative patients in group C

Variable	COVID-19 positive (*n* = 34)	COVID-19 negative (*n* = 242)	*p* value
Age, years, mean (SD)	85.0 (7.7)	80.8 (11.5)	0.043
Gender %, M:F	41.2:58.8	36.5:63.5	0.598
ASA, *n* (%)			
1	0	4 (1.7)	0.449
2	6 (17.7)	67 (27.7)	0.209
3	18 (52.9)	122 (50.4)	0.800
4	10 (29.4)	48 (19.8)	0.182
5	1 (2.9)	0	0.707
CCI, mean (SD)	5.4 (1.4)	5.2 (2.2)	0.355
NHFS, mean (SD)	5.8 (1.5)	4.8 (1.7)	0.001
Time to surgery, h, mean (SD)	54.8 (60.1)	42.1 (29.2)	0.423
Length of stay, days, mean (SD)	17.6 (8.9)	14.7 (10.8)	0.014
Mortality, *n* (%)	13 (38.2)	14 (5.8)	< 0.001

*SD* standard deviation, *ASA* American Society for Anaesthesiology, *M* male, *F* female, *CCI* Charlson Comorbidity Index, *NHFS* Nottingham Hip Fracture Score

## Discussion

This, to our knowledge, is one of the largest studies in the literature to date examining the impact of the COVID-19 pandemic on the management of hip fracture patients. The average number of hip fracture patients being admitted per day during the COVID-19 time period in this study was identical to the pre-COVID-19 time point. This reflects the fact that for these patients, falls at home or their usual place of residence is the commonest location when sustaining a hip fracture [27]; therefore, social distancing and isolation do not affect this. In an observational study in Spain, the number of hip fracture patients admitted during the COVID-19 pandemic time period was similar to other earlier time points [11].

The COVID-19 pandemic and subsequent hospital restructuring have had a major effect on time to theatre, length of hospital stay and 30-day mortality rate for COVID-19-positive hip fracture patients. The BOA guidance on managing trauma patients in the COVID-19 situation stated “care of patients with hip and femoral fractures remains urgent and a surgical priority.” [18]. Taking this into account, patients with an unknown status of COVID-19 needing surgery should ideally be managed as COVID-19-positive patients in ‘hot’ theatres to avoid delays in awaiting test results. However, this was difficult to put into practice, due to changes in operative procedures for COVID-19 positive patients, the extra staff required to run a ‘hot’ theatre and limited theatre capacity. This resulted in patients awaiting a minimum of 24 h for swab results prior to operating. Delays to theatre during the pandemic were also due to the following:

1. The requirement to undertake surgery in specific ‘hot’ (COVID-19 positive) and ‘cold’ (COVID-19 negative) theatres.
2. The use of full personal protective equipment (PPE) during aerosol-generating procedures (AGP) such as intubation and drilling, in order to reduce the risk of COVID-19 transmission. This inevitably meant longer operative (combined anaesthetic and surgical) times for patients.
3. The reduced availability of theatre capacity as many theatres were converted to intensive care facilities in preparation for the expected surge in COVID-19 patients needing ventilatory support.
4. The constantly changing guidelines for operating in the COVID-19 situation with regard to PPE and AGPs. Hospitals needed to adapt rapidly to an ever-changing environment, which led to significant challenges in theatre capacity and efficiency.
5. The time taken to wear and remove full PPE as well as the need to have additional staff in theatres with staff sickness and shielding reducing the available workforce.

There was a significant difference in the proportion of THRs and conservative management in the COVID-19 time period, reflecting the advice from the BOA to consider avoiding the more complex option of a THR in favour of doing a hemiarthroplasty for displaced intracapsular hip fractures [18]. Interestingly whilst in our study, 5% of patients in the COVID-19 era (11% in 2019) had a THR, in the London study by Kayani et al., the percentage of patients receiving a THR who were COVID-19 positive and negative was much higher at 12.2% and 10.9%, respectively [20]. The annual NHFD 2019 report found that less than 10% of patients with a displaced intracapsular hip fracture had a THR [12]. Similarly, more patients were treated non-operatively as the risks of surgery were deemed to outweigh the potential benefits for this select extremely vulnerable group of patients.

Although the CCI of the patients in the two groups was comparable, there were significantly more ASA 4 patients in group C (20.1%) compared to group PC (12.2%). The COVID-19-positive patients also had higher NHFS, reflecting their greater co-morbidities and risk of dying after hip fracture. A NHFS of ≤4 is considered low risk and a score of ≥5 high risk with a 30-day mortality of 3.5% vs 13.7%, respectively [28]. The reasons for this are not known, but it could be related to their delayed presentation or reduced availability of social care during the COVID-19 period. A larger national study may be required to test this hypothesis. There was also a longer time to theatre (which was trending towards statistical significance) in the COVID-19-positive patients. This is similar to other studies where the time to theatre was between 2 and 5 days [20, 29].

The NHFD has reported overall national 30-day mortality rates of approximately 6.4% for the time period of March to May 2019 [30]. In our study, the overall mortality rate for group C across the three units was 9.8%. Whilst the mortality rates for patients in group PC were similar to that of those in group C, within group C, COVID-19-positive patients were almost seven times more likely to die than COVID-19-negative patients. This reflects the seriousness of the COVID-19 infection and its sequelae in such elderly, vulnerable patients. There have been some small case series studies on the mortality rates of COVID-19-positive patients with hip fractures. In a retrospective study of 10 patients in New York, who were admitted with a hip fracture and were COVID-19 positive, one patient died at day 19 post-operatively (10% mortality rate) [31]. One of the earliest case series from China, looking at 10 patients with a hip fracture, one of the three patients who was subsequently found to be COVID-19 positive after their surgery, died [32]. Two studies from Italy have also reported that the mortality rates in this group of patients were 31% in a series of 16 cases [33] and 44% in a cohort of 32 patients within 21 days of surgery [34]. A multicentre, observational study in Spain found an early mortality rate of 30.4% in 136 hip fracture patients who were treated surgically or nonoperatively during the COVID-19 pandemic [29]. These studies would support our findings of a mortality rate of 35% in COVID-19-positive hip fracture patients.

The NHFD has reported overall acute hospital length of stay to be 15.1 days for the time period of March to May 2019 [30]. The hospital length of stay (LOS) for patients in group PC was significantly longer compared to LOS for patients in group C. This probably reflects a coordinated effort to discharge patients more quickly during the COVID-19 pandemic, in order to create sufficient bed capacity to cope with the anticipated influx of COVID-19-positive patients being admitted. There was a significantly longer hospital stay for COVID-19-positive patients compared to COVID-19-negative patients. COVID-19-positive patients may have required further clinical treatment, and care home facilities were reluctant to accept COVID-19-positive patients back, until they had been in isolation for the required period of time. A study conducted at a single NHS hospital trust comprising 157 hip fracture patients admitted in March to May 2020 showed similar findings; approximate length of hospital stay for COVID-19-negative patients was lower at 12 days vs 17 days, although not statistically significant [35]. Another multicentre UK study comprising 404 hip fracture patients admitted in March 2020 again showed similar findings; approximate length of hospital stay for COVID-19-negative patients was statistically significantly lower at 14 days vs 18 days [36].

This study has its limitations inherent in any retrospective study design. Data on delayed complication rates and revision rates of surgery were not available in this short time period analysed. Whilst every attempt has been made to check mortality rates using the ONS database with local hospital records, deaths occurring out of hospital may not have been identified because of the short follow-up period, which may have led to an underestimation of the mortality rate for these patients. In addition, differences in mortality rates across hospitals were observed. The reasons for this are unclear but are likely to be multi-factorial and may reflect variation in the incidence of COVID-19-positive patients in each region. Finally, adjusted analyses were not conducted to see how factors other than admission time period or COVID-19 diagnosis may have played a role in mortality rates. This study, however, has many strengths including its multi-centre design covering a large part of England outside of London. Nearly 600 consecutive patients were analysed with a widespread distribution of their pre-injury and peri-operative variables allowing a better comparison of their management during the COVID-19 and pre-COVID-19 periods.

## Conclusion

Despite the challenges COVID-19 has presented and even though hip fractures patients during the COVID-19 pandemic experienced a longer time to theatre, it is reassuring to note that for these patients, the 30-day mortality rate was similar to and the length of hospital stay shorter than patients admitted in the same time period prior to the pandemic.

However, when comparing patients who were COVID-19 positive to those who were COVID-19 negative during the pandemic, an even longer time to theatre, a significantly longer hospital length of stay and a significantly higher mortality rate were observed. This is possibly due to changes in pre-operative and operative procedures to treat COVID-19-positive patients [18, 37] and delays in receipt of COVID-19 test results.

Whilst we recommend maintaining strict protocols to minimise the risk for COVID-19 transmission, this population of patients requires urgent care. It may be useful to treat patients with an unknown COVID-19 status as COVID-19-positive patients when theatre room capacities allow for this to prevent surgical delays for patients with hip fractures.

## Data Availability

The datasets used and analysed during the current study are available from the corresponding author on reasonable request.

## Abbreviations

PC: Pre-COVID
C: COVID
ONS: Office for National Statistics
SARS-CoV-2: Severe acute respiratory syndrome coronavirus 2
UK: United Kingdom
BOA: British Orthopaedic Association
DGH: District general hospitals
MTC: Major trauma centre
RT-PCR: Reverse transcriptase-polymerase chain reaction
ASA: American Society of Anesthesiologists
SD: Standard deviations
CCI: Charlson Comorbidity Index
NHFS: Nottingham Hip Fracture Score
NHFD: National Hip Fracture Database
DHS: Dynamic hip screw
THR: Total hip replacement
M: Male
F: Female
IM: Intramedullary
ORIF: Open reduction internal fixation
CHS: Cannulated hip screws
PPE: Personal protective equipment
AGP: Aerosol-generating procedures
NHS: National Health System
